# Comparison of ^18^F-FDG PET/CT and ^68^Ga-FAPI in Spindle Cell Rhabdomyosarcoma

**DOI:** 10.3390/diagnostics13183006

**Published:** 2023-09-20

**Authors:** Zhihui Shen, Ruimin Wang

**Affiliations:** Department of Nuclear Medicine, The First Medical Centre, Chinese PLA General Hospital, No. 28 Fuxing Road, Beijing 100853, China; shenzhihui301@126.com

**Keywords:** ^18^F-FDG, ^68^Ga-FAPI, PET/CT, spindle cell rhabdomyosarcoma

## Abstract

We report a rare case of spindle cell rhabdomyosarcoma. Sarcomas generally exhibit an abnormal increased FDG uptake on ^18^F-FDG PET/CT imaging, while spindle cell rhabdosarcomas exhibits a significantly increased lesion uptake on ^68^Ga FAPI PET/CT imaging compared to ^18^F-FDG. This case suggests that ^68^Ga-FAPI PET/CT has potential value in evaluating spindle cell rhabdomyosarcoma.

The study and understanding of Rhabdomyosarcoma have been of immense importance due to its rarity and the challenges associated with its diagnosis and treatment. Over the years, advancements in medical imaging and diagnostic techniques have played a pivotal role in detecting and treating this malignancy. Rhabdomyosarcoma is a rare type of cancer that is mostly found in children. Current research has seen significant advancements in the diagnosis and treatment of this malignancy. Rhabdomyosarcoma is a rare soft tissue tumor, but it is relatively common in children. Rhabdomyosarcoma accounts for 4–8% of malignant soft tissue tumors in children, most of which originate from muscularly rich areas such as the head and neck, limbs, trunk, buttocks, and scapulae, and are most likely to occur in the limbs, followed by the head and neck [1,2]. Rhabdomyosarcoma is classified as embryonal, acinar, and pleomorphic rhabdomyosarcoma [3].

Spindle cell rhabdomyosarcoma (SCRM) originally belonged to the subtype of embryonal rhabdomyosarcoma, but in the WHO classification of soft tissue tumors (2013 edition), SCRM and sclerosing rhabdomyosarcoma were separately combined into a category called spindle cell/sclerosing rhabdomyosarcoma [4]. SCRM is most common in children and adolescents, with a tendency to occur in males [5]. It has this characteristic in both children and adults, with an onset age of 0.3 to 79 years [6,7]. SCRM has a good prognosis in children, but a poor prognosis in the adult population [8].

^18^F-FDG PET/CT can reflect the glucose metabolism of tumors and play an important clinical role in the postoperative and post-treatment evaluation of SCRM, which can detect tumor recurrence early. Secondly, PET/CT imaging helps to understand the overall situation, detect metastasis in the early stage, and exclude other tumor lesions, which has high clinical value [9]. FAPI is a type II membrane-binding glycoprotein and a novel tracer [10]. There are no relevant literature reports on ^68^Ga-FAPI PET/CT imaging of SCRM. Therefore, we introduced a series of SCRM with combined ^18^F-FDG PET/CT and ^68^Ga-FAPI PET/CT PET/CT imaging.

A 62-year-old male patient had no prior family history of tumor or surgical treatment. The patient has been in good health without any discomfort. The patient was found to have a left posterior thigh mass without obvious inducement 4 months prior, and the mass was found to be significantly larger than 2 months prior. Later, he went to the general surgery department of our hospital. Laboratory tests of the patient were normal. A malignant tumor was suspected. The general surgeon recommended that the patient use PET/CT to detect primary tumors and participated in a comparative clinical trial of ^18^F-FDG and ^68^Ga-FAPI PET/CT in malignant solid tumors approved by the Institutional Review Committee (S2022-750-01). ^18^F-FDG PET-CT was performed using the Siemens Biograph 64 PET/CT system. The patients fasted for at least 6 h and rested in a quiet environment for at least 20 min. The height, weight, and fasting blood glucose level were measured before scanning. Blood glucose levels were controlled within 6.5 mol/L. Prohibit oral administration of drugs that may affect FDG intake, such as glucose, amino acids, and nutrient solutions. After the intravenous injection of ^18^F-FDG (4.44–5.55 MBq/kg [0.12–0.15 mCi/kg]), the ^18^F-FDG PET/CT images were collected after a rest period of 58 ± 7.1 (SD) min. CT for PET/CT was performed as low-dose CT without any IV or oral contrast medium. CT was performed first, and then PET was performed from the femur to the skull base. Four or five PET beds were routinely used. The tube current of CT was 110 mAs, the voltage was 120 KV, the rotation time of the tube was 0.5 s, the layer thickness was 5 mm, and the pitch was 1. The PET acquisition time was 2 min/bed using CT-based attenuation correction and the point spread function to correct the reconstruction. The reconstruction parameters were as follows: 3 iterations, 21 subsets, 256 mm × 256 mm matrix, 5.0 mm FWHM Gaussian postfiltering, the voxel size of the reconstructed image (1.65 mm, 1.65 mm, 3 mm), and the scatter correction. ^18^F-FDG PET/CT scan showed hypermetabolic lesions in the posterior muscle layer of the left lower thigh, with an uneven metabolism. The maximum standard uptake value (SUVmax) was 6.8, and necrotic areas were visible within the lesion (Figure 1). The CT scan of the same machine showed a soft tissue density mass in the posterior muscle layer of the left lower thigh, with uneven density and a size of about 7.8 × 9.9 × 14.3 cm. No strong uptake was observed elsewhere in the patient that might present as a primary tumor. Based on the ^18^F-FDG PET/CT findings, primary malignant tumors are considered, and sarcoma is highly suspected. The patient underwent ^68^Ga-FAPI PET/CT imaging 2 days later. The same Biograph 64 PET/CT equipment (Siemens, Germany) was used, and the acquisition and reconstruction parameters remained unchanged. Image acquisition was performed after intravenous injection of 185 MBq (5mCi) ^68^Ga-FAPI for 60 min. ^68^Ga-FAPI PET/CT scan showed abnormal and uneven FAPI uptake in the posterior muscle layer of the left lower thigh, SUVmax: 11.3 (Figure 2). ^68^Ga-FAPi-derived SUVmax was significantly higher than ^18^F-FDG PET/CT (11.3 vs. 6.8). This patient combined with two tracer imaging was considered sarcoma. Finally, the patient underwent an ultrasound-guided biopsy of the lesion, and pathological examination showed spindle cell rhabdomyosarcoma in the posterior muscle layer of the left lower thigh (Figure 3). Immunohistochemical results: CD34 (vascular+), CD31 (vascular+), Vimentin (+), EMA (−), S-100 (−), SMA (−), Ki-67 (+50%), MUC4 (−), SOX-10 (−), Myo-D1 (partially+), and Desmin (+).

Finally, the patient underwent surgery in a local hospital. Telephone follow-ups were conducted for the following 2 years, and no recurrence or metastasis was found in the patient’s local hospital.

This study on spindle cell rhabdomyosarcoma imaging illuminates the crucial need for tailored diagnostic approaches, especially for rare tumors predominantly found in children. Highlighting the potential of ^68^Ga-FAPI PET/CT as a superior alternative to the conventional ^18^F-FDG PET/CT, the research not only promises enhanced patient care through clearer tumor visualization but also bridges a notable gap in medical imaging. The innovative introduction of this novel imaging technique suggests a potential shift in diagnostic paradigms, advocating for a more tumor-specific imaging approach. By providing empirical evidence through a demonstrative case, the study paves the way for future clinical trials and potentially transformative changes in the diagnostic protocols for spindle cell rhabdomyosarcoma.

Spindle cell rhabdomyosarcoma (SCRM) are rare tumors forming only 5–10% of all rhabdomyosarcoma cases and are now considered a distinct entity, separate but related to embryonal rhabdomyosarcoma. There is a male preponderance with a male-to-female ratio of 6:1 [10]. SCRM has a good prognosis in children, but a poor prognosis in the adult population [11]. The 5-year survival rate in children is 95%, while the recurrence and metastasis rate in adults is 40% to 50% [12]. Therefore, the early diagnosis and treatment of SCRM is very important for the prognosis of patients.

Computed tomography (CT) and magnetic resonance imaging (MRI) serve as the routine means for local relapsed surveillance. But for detecting distant metastasis, 18F-fluorodeoxyglucose (^18^F-FDG) positron emission tomography/computed tomography (PET/CT) shows higher sensitivity and accuracy [13]. Furthermore, ^18^F-FDG PET/CT is useful for initial staging and restaging, evaluation of treatment response, and predicting treatment efficacy and clinical outcome for soft tissue sarcomas [14]. However, due to a lack of sensitivity among some subtypes of sarcomas, particularly low-grade sarcomas, ^18^F-FDG PET/CT is not generally recommended for sarcomas [15].

Recently, the new development of PET tracers targeting fibroblast activation protein (FAP), ^68^Ga-fibroblast activation protein inhibitor (^68^Ga-FAPI), had shown promising results in the imaging of sarcomas [16]. FAP is a type II membrane-bound glycoprotein belonging to the dipeptidyl peptidase 4 family, which has both dipeptidyl peptidase and endopeptidase activity. FAP plays a pivotal role in the tumor microenvironment, including reduced levels of anti-angiogenic factors, elevated levels of transforming growth factor β, and affected matrix processing enzymes [17]. FAP is overexpressed in cancer-associated fibroblasts (CAFs) in the stroma of more than 90% of epithelial carcinomas and many subtypes of soft tissue sarcomas (e.g., fibrosarcoma, malignant fibrous histiocytoma, and liposarcoma) [18]. In addition to diagnostic imaging, FAP is also considered a promising target for delivering therapeutic nuclide [19]. This may provide a new approach for recurrent SCRM to improve survival. Thus, the expression of FAP on SCRM needs to be identified. In previous studies [20], ^18^F-FDG PET/CT detected approximately two-thirds of recurrent lesions with a sensitivity of 65.96%, a specificity of 21.43%, and an accuracy of 61.94% in the 13 subtypes of soft tissue sarcomas. Compared to ^18^F-FDG, ^68^Ga-FAPI PET/CT identified almost all lesions (275/282) and presented significantly improved sensitivity, specificity, and accuracy (97.52%, 60.71%, and 95.15%, respectively). In addition, the higher sensitivity and accuracy of ^68^Ga-FAPI over ^18^F-FDG PET/CT could bring benefits in accurately restaging and guiding the treatment decision in recurrent soft tissue sarcomas [21]. In this case, ^68^Ga-FAPI PET/CT showed significantly FAPI higher uptake in SCRM patients than ^18^F-FDG, which is in line with previous studies [22].

It should be noted that the intensive uptake of ^68^Ga-FAPI presenting in wound healing, uterus, arthritis, and periodontitis may be misdiagnosed as local relapse or distant metastasis. This is caused by fibrotic activity in these conditions [23]. Thus, more examples of research focused on the non-tumor-specific uptake of FAPI are still needed [24]. 

Despite advances in chemotherapy, targeted therapy and immunotherapy over the last decades, the prognosis for patients with metastatic soft-tissue sarcomas remains poor [25]. Ferdinandus et al. [26] demonstrated the potential role of FAP-targeted radioligand therapy in a study of nine patients with solid tumors. Surprisingly, disease control was observed in three patients with sarcomas and one patient with pancreatic ductal adenocarcinoma. These studies indicated that FAP-targeted radioligand therapy may present as a novel promising treatment strategy for incurable recurrent soft tissue sarcomas. 

In summary, SCRM is an extremely rare malignant tumor. They need an early, accurate diagnosis and clear management plans. Early surgical interventions can reduce recurrence and lead to ideal results. In this case, ^68^Ga-FAPI PET/CT showed that significantly higher uptake of patients with SCRM than ^18^F-FDG. This case suggests that ^68^Ga FAPI PET/CT imaging may have important clinical significance for the diagnosis of SCRM.

## Figures and Tables

**Figure 1:**
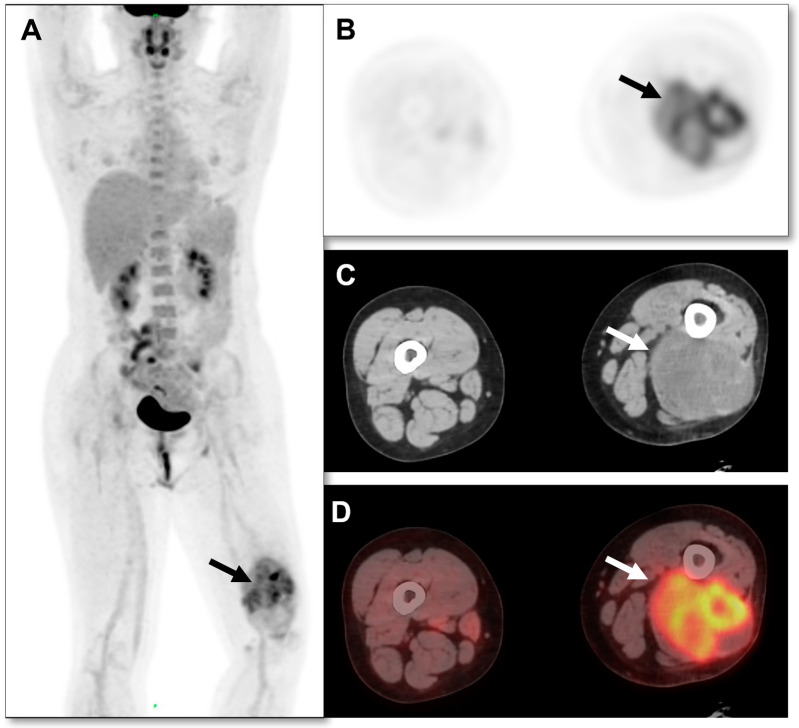
The maximum-intensity-projection (MIP) image of ^18^F-FDG PET/CT (**A**) showed increased radiotracer uptake in the posterior muscle layer of the left lower thigh (arrow). The MIP image does not show the presence of nodal or distant metastases. Axial images of PET (**B**), CT (**C**), and PET/CT fusion (**D**) showed soft tissue density mass in the posterior muscle of the left lower thigh with increased diffuse FDG radiotracer uptake and uneven distribution of lesion density and FDG (arrow; SUVmax 6.8; Size, 7.8 × 9.9 × 14.3 cm). No clear abnormal high radiotracer uptake was observed in other parts of the patient. Based on ^18^F-FDG PET/CT findings, the lesion was considered a primary malignant tumor.

**Figure 2:**
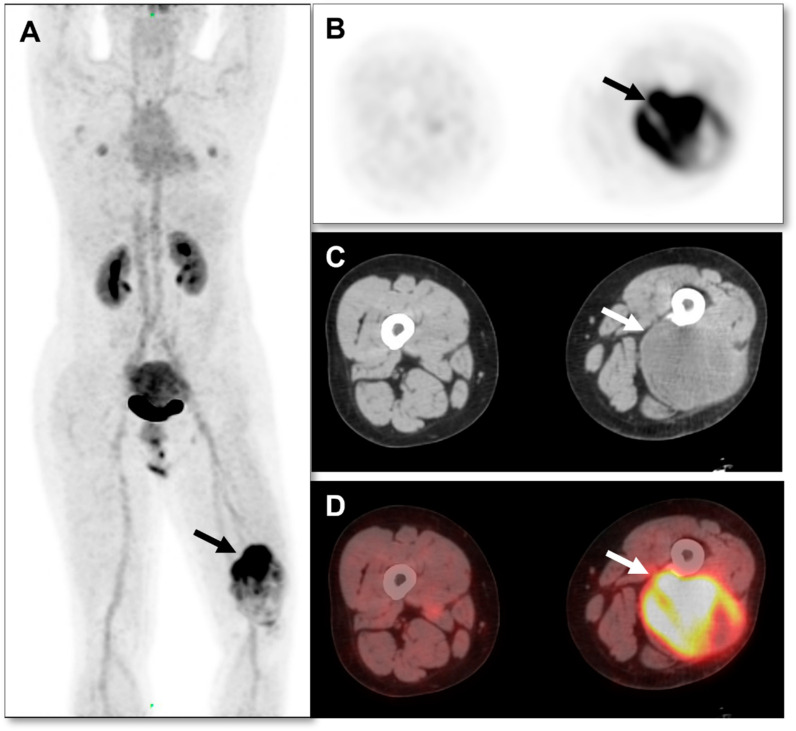
^68^Ga-FAPI PET/CT was performed 2 days after ^18^F-FDG PET/CT. ^68^Ga-FAPI PET/CT MIP image (**A**), arrow indication showing abnormal ^68^Ga-FAPI high radiotracer uptake mass in the posterior muscle of the left lower thigh, SUVmax: 11.3. MIP image does not show lymph nodes or distant metastases. It is evident from the axial images ((**B**), CT; (**C**), PET; (**D**), fusion PET/CT) that FDG uptake is overall weak and more homogeneously distributed than ^68^Ga-FAPI, which conversely has a large focus of high uptake in the cranial half of the mass and a weaker uptake in the remainder. ^68^Ga-FAPI-derived SUVmax was significantly higher than ^18^F-FDG PET/CT (11.3 vs. 6.8, arrow indication). No significant abnormal FAPI radiotracer uptake was observed in other parts of the patient. This patient was considered for sarcoma with FDG and FAPI imaging.

**Figure 3:**
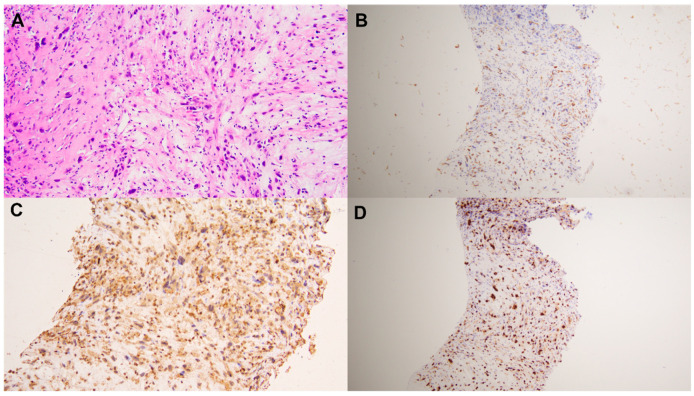
The patient underwent an ultrasound-guided biopsy of the lesion, and pathological examination showed spindle cell rhabdomyosarcoma in the posterior muscle layer of the left lower thigh ((**A**), hematoxylin and eosin staining, original magnification ×40). Immunohistochemical examination showed positive staining for Vimentin ((**B**), original magnification ×10), Desmin ((**C**), original magnification ×10), and Ki-67 + 50% ((**D**), original magnification ×10).

## Data Availability

The data are not publicly available due to privacy regulations regarding patients.
